# Prevalence and Correlates of Preference-Concordant Care Among Hospitalized People Receiving Maintenance Dialysis

**DOI:** 10.34067/KID.0000000000000131

**Published:** 2023-05-05

**Authors:** Basil S. Kazi, Paul R. Duberstein, Benzi M. Kluger, Ronald M. Epstein, Kevin A. Fiscella, Zain S. Kazi, Spencer K. Dahl, Rebecca J. Allen, Fahad Saeed

**Affiliations:** 1 Department of Internal Medicine, University of Illinois at Chicago, Chicago, Illinois; 2 School of Medicine and Dentistry, University of Rochester, Rochester, New York; 3 Department of Health Behavior, Society and Policy, Rutgers School of Public Health, Piscataway, New Jersey; 4 Department of Palliative Care, University of Rochester Medical Center, Rochester, New York; 5 Department of Neurology, University of Rochester Medical Center, Rochester, New York; 6 Department of Family Medicine, University of Rochester Medical Center, Rochester, New York; 7 Department of Public Health Sciences, University of Rochester Medical Center, Rochester, New York; 8 Institute of Advanced Analytics, North Carolina State University, Raleigh, North Carolina; 9 School of Behavioral and Natural Sciences, Mount St. Joseph University, Cincinnati, Ohio; 10 Department of Nephrology, University of Rochester Medical Center, Rochester, New York

**Keywords:** preference-concordant care, goal-concordant care, shared decision-making, dialysis decision-making, palliative care, symptoms, dialysis, hospitalization, prognosis, end-stage kidney disease, geriatric and palliative nephrology

## Abstract

**Key Points:**

- A large proportion of hospitalized patients receiving dialysis report not receiving preference-concordant care.
- Hospitalized patients on dialysis desiring a comfort-oriented medical plan were likely to report receiving preference-concordant care.

**Background:**

Preference-concordant care is a cornerstone of high-quality medical decision-making, yet the prevalence and correlates of preference-concordant care have not been well-studied in patients receiving dialysis. We surveyed hospitalized people receiving maintenance dialysis to estimate the prevalence and correlates of preference-concordant care among this population.

**Methods:**

We assessed preference concordance by asking participants (223/380, 59% response rate), “How strongly do you agree or disagree that your current treatment plan meets your preference?” We assessed treatment plan preference by asking whether patients preferred a plan that focused on (*1*) extending life or (*2*) relieving pain and discomfort. We assessed shared dialysis decision-making using the 9-item Shared Decision-Making Questionnaire. We examined the differences between those reporting lack of preference-concordant care and those reporting receipt of preference-concordant care using chi-squared analyses. We also studied whether patients' treatment plan preferences or shared dialysis decision-making scores were correlated with their likelihood of receiving preference-concordant care.

**Results:**

Of the 213 respondents who provided data on preference concordance, 90 (42.3%) reported that they were not receiving preference-concordant care. Patients who preferred pain and discomfort relief over life extension were less likely (odds ratio, 0.15 [95% confidence interval, 0.08 to 0.28] *P* = <0.0001) to report receiving preference-concordant care; patients with higher shared decision-making scores were more likely (odds ratio, 1.02 [95% confidence interval, 1.01 to 1.03], *P* = 0.02) to report preference-concordant care.

**Conclusions:**

A substantial proportion of this sample of hospitalized people receiving maintenance dialysis reported not receiving preference-concordant care. Efforts to improve symptom management and enhance patient engagement in dialysis decision-making may improve the patients' perceptions of receiving preference-concordant care.

## Graphical abstract

**Figure:**
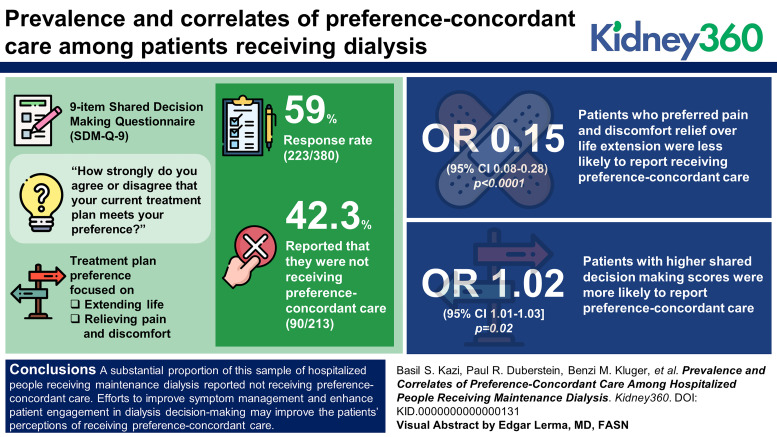

## Introduction

Preference-concordant care entails incorporating patients' values, goals, and preferences into their treatment plan.^1^ Delivery of preference-concordant care is vitally important for patients with ESKD receiving maintenance dialysis given their high mortality, excessive symptom burden, and poor quality of life.^1–5^ Patients with ESKD are hospitalized on average of almost twice a year with each hospitalization portending increased risks of mortality and debility among these patients.^5–7^ While hospitalization can be an excellent opportunity to reassess patients' treatment preferences and align them with their medical care,^8^ it is currently unknown to what extent hospitalized patients receiving maintenance dialysis report preference-concordant care.

Previous studies have shown that the focus of patients' treatment plan preferences can determine their likelihood of receiving preference-concordant care.^9,10^ Namely, in both inpatient and outpatient settings, patients who desire a comfort-oriented approach, as opposed to a life-prolonging approach, are less likely to receive preference-concordant care.^9,10^ In fact, only few patients are offered a comfort-oriented approach as an acceptable option. In one study, one of 99 patients receiving dialysis recalled talking with a clinician about a palliative or conservative approach to kidney management.^11^ Similarly, other studies suggest that many patients receiving dialysis are not offered a comfort-oriented approach.^12,13^

One plausible approach to provide preference-concordant care is by incorporating shared decision-making (SDM) principles into dialysis decisions.^14,15^ SDM is a process that involves patients and their families in making medical decisions according to patient treatment preferences.^16^ Previous studies in nondialysis populations have shown benefits of SDM, including improved satisfaction with health care decision-making and higher patient activation after the decision-making process.^17–19^ Such positive long-term effects of SDM on patient self-efficacy suggest that SDM could potentially be associated with perceptions of receiving preference-concordant care after dialysis initiation even among hospitalized patients.

Based on the vital importance of preference-concordant care for persons receiving maintenance dialysis, especially those who are acutely ill and hospitalized,^8^ the relative lack of support for patients preferring a comfort-oriented approach,^9,10,20^ and the possible association of SDM during dialysis decision-making with a subsequent perception of receiving preference-concordant care,^18, 21–23^ this study has three aims: (*1*) to estimate the proportion of hospitalized patients receiving maintenance dialysis who do not perceive their treatment as preference-concordant, (*2*) to test the hypothesis that patients who prefer a plan of care that prioritizes relieving pain and discomfort are less likely to perceive their treatment plan as preference-concordant than patients who prioritize extending life; and (*3*) to test the hypothesis that better SDM during the dialysis decision-making process is associated with improved preference concordance even after dialysis initiation. To the best of our knowledge, no previous study has examined these questions for hospitalized patients receiving maintenance dialysis.

## Methods

We surveyed hospitalized patients receiving maintenance dialysis from 06/2019 to 03/2020 at Strong Memorial Hospital in Rochester, New York. Using convenience sampling methodology, we identified eligible participants using an electronic medical record list of hospitalized patients receiving maintenance hemodialysis before hospitalization. We screened patients who were aged 18 years or older and were English-speaking. Subsequently, a survey team member (BK or SD) approached a patient's health care team member to determine whether the clinical team believed that the patient had capacity to consent. If so, a member of the study team approached eligible participants. We did not exclude patients based on their location (intensive care unit versus medical ward), length of hospitalization, reason for hospitalization, or comorbidities. Study team members (BK, SD) explained the purpose of the study to the patient, asked the patient to explain back the purpose of the study and their role in the study, and then asked for and obtained verbal consent. All patients who consented to the study were given the option to either complete a paper survey or respond to orally administered survey questions. The 175 survey items covered participants' demographics, views on their dialysis decision-making process, and psychosocial aspects of their care. All participants had the option to skip any questions or stop the survey at any point for any reason. A physician was also available to speak with patients in case of psychological distress. Patients who completed the survey were offered a chance to enter a raffle to win one of four $50 gift cards. The Institutional Review Board at the University of Rochester Medical Center approved this study.

### Assessments

**The primary outcome variable** for this study is preference-concordant care. To assess preference-concordant care, we asked patients: “How strongly do you agree or disagree that your current treatment plan meets your preference?” Response options included (*1*) strongly disagree, (*2*) disagree, (*3*) slightly disagree, (*4*) slightly agree, (*5*) agree, or (*6*) strongly agree. Any participant who responded slightly agree, agree, or strongly agree was deemed to be receiving preference-concordant care. Consistent with the previous literature, we assessed preference concordance after first assessing patients' treatment plan preferences (comfort-oriented versus life prolongation; see below).^9,10,24,25^ We specifically used this approach to prime patients to identify their treatment plan preferences before reflecting on whether their treatment plan meets these preferences. Consistent with the previous research evaluating patients' receipt of preference-concordant care, we intentionally did not state to the participants whether our questions were regarding their acute hospitalization or overall plan of care.^9,10,24,25^ This methodology provides us the benefit of capturing patients' assessment of the aspect of medical care most important to them.^9^

**The two independent variables** were treatment plan preferences and SDM. To understand patients' treatment plan preferences, we asked patients the following question^26^: “If you had to make a choice at this time, would you prefer a plan of medical care that (*1*) focuses on extending life as much as possible, even if it means having more pain and discomfort, or (*2*) would you want a plan of medical care that focuses on relieving pain and discomfort as much as possible, even if that means not living as long?” While many patients may prefer both options, we offered only two mutually exclusive options to encourage respondents to pick their top priority.^25^ We used the 9-item Shared Decision-Making Questionnaire (SDM-Q-9) to assess SDM during the process of dialysis decision-making.^27^ This 9-item, internally consistent (*α*=0.93) questionnaire gives patients the option to completely disagree, strongly disagree, somewhat disagree, somewhat agree, strongly agree, or completely agree with statements such as “My doctor and I selected a treatment option together.” Patients were informed that each statement in the questionnaire specifically referred to the decision-making process that led them to receive dialysis. A score of zero through five for each question was recorded and summed to determine a raw score of 0–45. Consistent with the previous literature, we then multiplied this score by 20/9 to calculate an SDM-Q-9 score from 0 to 100, where the scores of 0 and 100 describe the lowest and highest levels of SDM, respectively.^27,28^

**The covariates** included demographic data, financial strain, relationship status, time on dialysis, and advance care planning documentation (ACP). Demographic data included age, sex (woman versus man versus nonbinary), race (asked in the following order: White versus Black versus Asian versus Native Hawaiian or Pacific Islander versus American Indian versus Alaska Native versus Other), education, and income. Financial strain was measured using a set of four yes/no questions^29^ concerning respondents' beliefs that their income was enough for: (*1*) food and housing costs, (*2*) clothing, medicine, repairs to home, and transportation, (*3*) going out for a meal or entertainment, and (*4*) ability to take a vacation if their health permitted. Financial strain was considered present if participants responded yes to two or more questions.^29^ Relationship status was measured by asking patients whether they were married, divorced, widowed, separated, in a committed relationship, or single and never married. Patients were determined to be in a relationship if they reported they were married or were in a committed relationship. Time on dialysis was measured by asking patients, “How long have you been undergoing dialysis?” ACP was assessed by asking patients if they had a health care proxy, had a living will, or completed a Medical Orders for Life-Sustaining Treatment form. Advance care planning was considered present if the respondent had completed at least one of the three ACP documents.

### Statistical Analyses

All statistical analyses were conducted using SAS 9.4. Race was categorized as White versus Black versus Other, education as at least some college or technical school versus high school or below, and income as ≤$20,000 versus >$20,000. No participant identified as nonbinary leading to a categorization of sex as women versus men. We applied chi-squared testing to examine the differences between those reporting lack of preference-concordant care (strongly disagree, disagree, or slightly disagree) versus those reporting receipt of preference-concordant care (slightly agree, agree, or strongly agree). We then built a multiple logistic regression model for hypothesis testing by including variables with a *P*-value of <0.1 in the bivariate analyses. Ten respondents did not answer the question regarding preference-concordant care and were excluded from all statistical analyses. Of the remaining surveys, we found eight missing responses for age and sex, six for race and income, and ten missing responses for education. Forty-three respondents had missing responses for relationship status, seven for financial strain, and 15 for time of dialysis dependency. We did not find any missing responses for items on advance care planning, treatment plan preferences, and SDM. For the multiple logistic regression, the missing data for each of these variables were imputed over 15 iterations using the fully efficient fractional imputation method to reduce variability and effects on subsequent statistical analysis.^30^ We report odds ratios (OR), 95% confidence intervals (CIs), and the *P*-values in the bivariate analysis and the multiple logistic regression analysis as (OR [95% CI], *P*-value). Findings in the multiple logistic regression were deemed to be statistically significant if *P*-value was ≤0.05.

We conducted additional sensitivity analyses by (*1*) building two separate logistic regression models for each independent variable, (*2*) building a stepwise regression model by including all the variables, and (*3*) building an ordinal logistic regression model by grouping the six response options of the question related to preference-concordant care into three ordinal variables (strongly disagree combined with disagree, slightly disagree combined with slightly agree, and agree combined with strongly agree). We conducted these sensitivity analyses using the same imputation method described for main model.^30^

## Results

We approached 380 hospitalized patients receiving dialysis who met the eligibility criteria, of whom 223 participated (response rate: 58.7%). Of these 223 respondents, 213 responded to the questions on preference concordance and were included in our analyses. We did not purposely exclude people receiving peritoneal dialysis, but our sample comprised only of people receiving hemodialysis, likely because of a relatively lower number of patients receiving peritoneal dialysis in the Greater Rochester Area. Table 1 reports the respondents' characteristics: 116 (56.6%) were aged 65 or older, 103 (50.2%) women, 88 (42.5%) White, 117 (56.5%) made ≤$20,000 annually, and 102 (50.2%) had received no college education.

**Table 1:** Demographic characteristics of respondents

Patient Characteristic	*n* (%)
Total^a^	213
**Age (yrs)**	
Younger than 65 yrs	89 (43.4)
65 or older	116 (56.6)
**Sex** ^b^	
Women	103 (50.2)
Men	102 (49.8)
**Race**	
White	88 (42.5)
Black	82 (39.6)
Other (Asian, Native Hawaiian or other Pacific Islander, American Indian or Alaska Native, Other)	37 (17.9)
**Income**	
≤$20,000	117 (56.5)
>$20,000	90 (43.5)
**Education**	
High school or less	101 (49.8)
Some college or more	102 (50.2)
**Financial strain**	
Yes	127 (61.7)
No	79 (38.3)
**Relationship status**	
Married or in a committed relationship	127 (61.7)
Single, divorced, widowed, or separated	79 (38.3)
Mean years on dialysis (SD)	3.2 (0.6)
**Advance care planning**	
Health care proxy and/or MOLST	168 (78.9)
Unsure/none	45 (21.1)
**Preference concordance**	
Yes	123 (57.7)
No	90 (42.3)
**Treatment plan preferences**	
Relieving pain and discomfort	108 (50.7)
Extending life	105 (49.3)
Mean SDM-Q-9 score (SD)	57.6 (12.4)

MOLST, Medical Orders for Life-Sustaining Treatment; SDM-Q-9, 9-item Shared Decision-Making Questionnaire.

a Of all responses, we found the following missing data: eight missing responses each for the questions on age and sex, six each for race and income, ten for education, seven for financial strain, 43 for relationship status, and 15 for dialysis duration.

b Patients were given the option to identify as nonbinary. All respondents to this question identified as a woman or a man.

One hundred and twenty-three patients (57.7%) reported that their current treatment plan was preference-concordant, while 90 (42.3%) reported their treatment plan was not preference-concordant. One hundred twenty-three patients (57.7%) reported that if they had to choose at this time, they would prefer a plan of medical care that focused on relieving pain and discomfort as much as possible, even if that meant possibly living for less time than they might on dialysis. Ninety patients (42.3%) preferred a treatment plan that focused on extending life as much as possible, even if it meant having more pain and discomfort.

Table 2 describes the findings of the bivariate analysis. Patients who preferred a treatment plan that focused on relieving pain and discomfort were less likely to report preference-concordant care (OR, 0.14 [95% CI, 0.08 to 0.26] *P* = <0.0001), and patients who scored higher on the SDM-Q-9 scale were more likely to report preference-concordant care (OR, 1.02 [95% CI, 1.01 to 1.03] *P* = 0.003). Other variables with a *P*-value of <0.1 included educational attainment beyond high school (OR, 1.67 [95% CI, 0.95 to 2.93] *P* = 0.07), married or in a committed relationship (OR, 1.74 [95% CI, 0.93 to 3.23] *P* = 0.08), and the presence of an advance directive (OR, 2.20 [95% CI, 0.97 to 5.01] *P* = 0.06).

**Table 2:** Bivariate analyses independent variables and covariates in relation with preference concordance using chi-squared tests and logistic regression

Patient Characteristic	Do You Agree or Disagree That Your Current Treatment Plan Meets Your Preference Indicated Above? *n* (%)		Unadjusted OR (95% CI)	*P*-Value
Total patient cohort=213	Agree	Disagree		
	123 (57.7)	90 (42.3)		
**Treatment preferences**				
Comfort (ref)	39 (31.7)^a^	69 (76.7)^a^	0.14 (0.08 to 0.26)^a^	<0.0001^a^
Life extension	84 (68.3)^a^	21 (23.3)^a^
Mean SDM-Q-9 score (SD)	62.2 (11.7)^a^	50.8 (13.1)^a^	1.02 (1.01 to 1.03)^a^	0.003^a^
**Age**				
65 or older (ref)	65 (54.6)	51 (59.3)	0.83 (0.47 to 1.45)	0.50
Younger than 65 yrs	54 (45.4)	35 (40.7)
**Sex**				
Woman (ref)	57 (47.9)	46 (53.5)	0.80 (0.46 to 1.39)	0.43
Man	62 (52.1)	40 (46.5)
**Race**				
White	56 (46.3)	32 (37.2)	1.45 (0.83 to 2.56)	0.19
Black	46 (38.0)	36 (41.9)	0.85 (0.48 to 1.50)	0.58
Other	19 (15.7)	18 (20.9)		
**Income**				
≥$20,000 (ref)	53 (43.8)	37 (43.0)	1.03 (0.59 to 1.80)	0.91
<$20,000	68 (56.2)	49 (57.0)		
**Educational attainment**				
Some college or more (ref)	65 (55.1)^a^	36 (42.4)^a^	1.67 (0.95 to 2.93)^a^	0.07^a^
High school or less	53 (44.9)^a^	49 (57.6)^a^
**Financial strain**				
Present (ref)	79 (65.8)	48 (55.8)	1.53 (0.86 to 2.69)	0.15
Absent	41 (34.2)	38 (44.2)
**Relationship status**				
Yes (ref)	50 (51.0)^a^	27 (37.5)^a^	1.74 (0.93 to 3.23)^a^	0.08^a^
No	48 (49.0)^a^	45 (62.5)^a^
Mean years on dialysis (SD)	2.92 (0.5)	3.20 (0.6)	1.03 (0.91 to 1.15)	0.68
**Advance care planning**				
Present (ref)	112 (91.1)^a^	74 (82.2)^a^	2.20 (0.97 to 5.01)^a^	0.06^a^
Absent	11 (8.9)^a^	16 (17.8)^a^

OR, odds ratio; CI, confidence interval; SDM-Q-9, 9-item Shared Decision-Making Questionnaire.

a Significance <0.1.

Table 3 reports the findings of the multivariable logistic regression model. Patients who reported they preferred a treatment plan that focused on relieving pain and discomfort as much as possible were less likely to report preference-concordant care (OR, 0.15 [95% CI, 0.08 to 0.28] *P* = <0.0001). Notably, patients with higher SDM scores were more likely to report preference-concordant care (OR, 1.02 [95% CI, 1.01 to 1.03], *P* = 0.02). Educational attainment, relationship status, and the presence of and advance directive were not statistically significant in this model.

**Table 3:** Final adjusted multivariable logistic regression model of preference-concordant care (*N*=213)

Patient Characteristic		Adjusted OR Estimate	95% CI	*P*-Value
Prioritized plan that focused on relieving pain and discomfort		0.15^a^	0.08 to 0.28^a^	<0.0001^a^
SDM-Q-9 score		1.02^a^	1.01 to 1.03^a^	0.02^a^
Some college education or more		1.08	0.56 to 2.08	0.81
In relationship		1.37	0.67 to 2.79	0.39
Present advance care planning		1.77	0.83 to 3.80	0.14

OR, odds ratio; CI, confidence interval; SDM-Q-9, 9-item Shared Decision-Making Questionnaire.

a Significance < 0.05

We ran bivariate and logistic regression analyses without imputing the missing variables. The results (data not shown) were broadly consistent with those reported in Table 3. The results of all sensitivity analyses including two separate logistic regression models for each independent variable, a stepwise regression model and an ordinal regression model (see Supplemental materials) were also broadly consistent with the findings reported in Table 3.

## Discussion

In this cohort of hospitalized people receiving maintenance dialysis, we found that over 40% reported that their current treatment plan did not align with their preferences. Notably, preference for a plan of medical care focused on relieving pain and discomfort was associated with lower odds, while higher shared dialysis decision-making scores were associated with greater odds of receiving preference-concordant care. To the best of our knowledge, this is the first study to examine the prevalence and correlates of preference-concordant care in hospitalized patients receiving maintenance dialysis.

This study has three main findings. First, a significant proportion of patients did not feel that they were receiving preference-concordant care. Such lapses in preference-concordant medical care are striking but not surprising. Importantly, the proportion of patients reporting a lack of preference concordance can vary depending on the method of measuring preference-concordant care.^9,10^ Compared with some other studies of chronically ill patients, the proportion of patients reporting a lack of preference concordance is somewhat higher in our study.^9,10^ For example, in one recent study of hospitalized patients with a serious illness (not just ESKD), only 22% reported a lack of preference-concordant care.^10^ One possible explanation for this apparent discrepancy could be that patients with ESKD in our study reported lower rates of preference-concordant care because of their disease complexity and high number of comorbidities.^31^ Another plausible explanation is that previous studies provided their participants with a neutral or uncertain option,^9,10^ which we intentionally did not provide, mainly to identify patients' top priority.^25^ Future mixed-method studies comparing and exploring preference concordance among hospitalized patients by comorbidity may be helpful.

Second, patients who preferred a plan of medical care focused on relieving pain and discomfort were less likely to report preference concordance than patients who desired a plan focused on extending life. This finding is consistent with previous literature showing a negative correlation between the preference for a comfort-oriented approach and preference concordance in patients with chronic illnesses,^9^ including those hospitalized.^10^ One explanation for this finding is that some health care providers may assume that patients receiving maintenance dialysis prefer more aggressive treatments, given that dialysis itself is a life-sustaining treatment.^32^ However, an exclusive focus on life extension may potentially deprive patients of good symptom control with the help of primary or specialist palliative care.^33^ Another explanation is that some health care providers may be less equipped to treat the complex symptoms associated with ESKD and dialysis, leaving these patients largely undertreated.^3,12^ It is also plausible that some patients wish to explore dialysis withdrawal but are not offered opportunities to engage in such conversations.^34,35^ Future studies may provide clarity on some of these questions.

Third, our data suggest that patients who had engaged in SDM with their nephrologists during the dialysis decision-making process in the past were more likely to report preference-concordant care. Our findings broadly support the extensive literature on the benefits of SDM in nondialysis populations ranging from improved patient satisfaction, higher self-efficacy, and better quality of life to reduced decisional conflict, less decisional regret, lower health care costs, and fewer emergency department visits.^18,19,23,36^ Although a positive association of SDM with preference-concordant care has been demonstrated in patients with cancer,^21,22^ our study adds to the literature by suggesting a similar association in hospitalized patients receiving maintenance dialysis. Future studies are needed to establish a causal relationship between SDM and preference-concordant care.

Our study has several limitations. We did not capture patients' comorbid conditions, reasons for hospitalization, length of hospitalization, severity of illness, understanding of illness, symptom burden, kidney transplantation status, or affective state. All these may have potentially influenced patient responses. We also recognize that our study's participants' interpretation of the SDM process for their dialysis decision is subject to recall bias, especially during an acute hospitalization, when symptom burden may be high. Another notable limitation is that although our question assessing preference concordance was consistent with previous literature,^9,10,24,25^ our choice did not reference specific medical care (long-term dialysis care versus acute hospitalization versus overall care, *etc.*) and may have potentially led to different interpretations of the question by different participants. While this methodology certainly has limitations, it still provides us the benefit of capturing patients' assessment of the focus of the aspect of medical care most important to them. Finally, we assessed patients' treatment plan preferences at one period in time, and treatment plan preferences may change with time.^37^ Future research assessing patients' treatment plan preferences at multiple time points would gather a more robust understanding of these preferences.

Our results have several implications. First, our findings encourage health care providers to explore patients' treatment preferences during hospitalization by engaging in timely goals-of-care conversations. These conversations offer vital opportunities to learn and incorporate patient preferences into their treatment plan and are associated with improved patient satisfaction, fewer hospitalizations, and lower health care costs.^38,39^ Second, our findings, like others,^40^ encourage improved treatment of symptoms for patients receiving dialysis. Patients who receive dialysis report a high symptom burden,^41^ and many desire health care that focuses on relief of symptoms and suffering.^42^ However, many patients' symptoms remain undertreated, and many report being unaware of palliative care services.^42^ Finally, our findings call to improve dialysis decision-making by incorporating principles and practice of SDM.^14^ Encouraging routine use of decision aids and decision coaching may be a helpful adjunct in clinical SDM.^43,44^ Training clinicians in primary palliative care skills around communication and decision-making holds some promise in improving SDM and delivering preference-concordant care to patients receiving dialysis.^33,45,46^ Development and implementation of educational interventions for clinicians to develop primary palliative care skills are much needed.^47,48^

In summary, many hospitalized people receiving dialysis report that their treatment plan does not align with their preferences. There is a critical need for more robust efforts to not only align patients' preferences with their treatment plans by engaging in goals-of-care conversations but also to adequately treat the symptom burden of hospitalized patients receiving maintenance dialysis, perhaps with support from palliative care services. Our findings also suggest further incorporating the principles and practices of SDM into the treatment plan of people with chronic kidney disease facing dialysis decisions.

## Supplementary Material

SUPPLEMENTARY MATERIAL

## Supplemental Material

Supplemental Analyses 1A. Multivariable logistic regression of preference-concordant care using treatment plan preferences as an independent variable (*n*=213).

Supplemental Analyses 1B. Multivariable logistic regression of preference-concordant care using SDM-Q-9 as an independent variable (*n*=213).

Supplemental Analyses 2. Ordinal regression model of preference-concordant care using treatment plan preferences and SDM-Q-9 as independent variables (*n*=213).

Supplemental Analyses 3. Stepwise logistic regression model of preference-concordant care (*n*=213).
