# Supplementary material for: Prevalence and Correlates of Preference-Concordant Care Among Hospitalized People Receiving Maintenance Dialysis
Source: Kidney360. 2023 May 5;4(6):e751–8. doi: 10.34067/KID.0000000000000131 (PMC10371368; doi:10.34067/KID.0000000000000131)
Supplement: SUPPLEMENTARY MATERIAL [file kidney360-4-e751-s001.pdf]

Supplemental Analyses 1A: Multivariable logistic regression of preference-concordant care using treatment plan preferences as an independent variable(n=213)

	Adjusted Odds Ratio	95% CI	P-value
**Prioritized plan that focused on relieving pain and discomfort**	**0.17**	**0.11-0.29**	**<0.0001**
Some college education or more	1.18	0.63-2.18	0.76
In a relationship	1.29	0.58-2.58	0.42
Presence of advance care planning	1.71	0.73-3.55	0.10

Supplemental Analyses 1B: Multivariable logistic regression of preference-concordant care using SDM-Q-9 as an independent variable (n=213)

	Adjusted Odds Ratio Estimate	95% CI	P-value
**SDM-Q-9 score**	**1.02**	**1.01-1.03**	**0.02**
Some college education or more	1.13	0.59-2.11	0.78
In a relationship	1.53	0.79-2.90	0.37
Presence of advance care planning	1.72	0.74-3.57	0.13

Supplemental Analyses 2: Ordinal regression model of preference-concordant care using treatment plan preferences and SDM-Q-9 as independent variables (n=213)

	Adjusted Odds Ratio Estimate	95% CI	P-value
**Prioritized plan that focused on relieving pain and discomfort**	**0.10**	**0.03-0.17**	**<0.0001**
**SDM-Q-9 score**	**1.02**	**1.01-1.03**	**0.02**
Some college education or more	1.10	0.76-1.58	0.71
In a relationship	1.32	0.70-2.32	0.25
Presence of advance care planning	1.79	0.95-3.72	0.11

a. The outcome variable, preference-concordant care was treated as an ordinal variable. Responses were grouped as followed: strongly disagree or disagree, slightly disagree or slightly agree, and agree or with strongly agree.

Supplemental Analyses 3: Stepwise logistic regression model of preference-concordant care (n=213)

	**Adjusted Odds Ratio Estimate**	**95% CI**	**P-value**
**Prioritized plan that focused on relieving pain and discomfort**	**0.15**	**0.08-0.28**	**<0.0001**
**SDM-Q-9 score**	**1.02**	**1.01-1.03**	**0.02**
Some college education or more	1.08	0.56-2.08	0.81
In a relationship	1.37	0.67-2.79	0.39
Presence of advance care planning	1.77	0.83-3.80	0.14

*We implemented the backward elimination approach to develop this stepwise logistic regression model
